# Using Images of Eyes to Enhance Green Brand Purchase Intentions Through Green Brand Anthropomorphism Strategies: The Moderator Role of Facial Expression

**DOI:** 10.3389/fpsyg.2020.568595

**Published:** 2020-10-02

**Authors:** Zelin Tong, Tingting Li, Jingdan Feng, Qin Zhang

**Affiliations:** ^1^Management School, Hainan University, Haikou, China; ^2^Business School, Central South University, Changsha, China

**Keywords:** anthropomorphism, eye effect, green trust, purchase intention of green products, anthropomorphic strategies

## Abstract

This study uses an experimental comparison to analyze the effects of anthropomorphic strategies that use images with eyes (vs. those without eyes) on consumers’ willingness to buy green products, as well as the mechanism of action. The study finds that concerning the anthropomorphic features of a product, anthropomorphic strategies containing images of eyes lead to more positive purchase intention for green products than those without images of eyes. Simultaneously, these green purchase intentions are mediated through the variable of green trust regardless of whether the anthropomorphic strategies feature eyes. Beyond this, the discussed effects are moderated when anthropomorphic strategies featuring different facial expressions are used. The findings of this study provide useful suggestions for green brand marketing strategies and management practices that use anthropomorphism.

## Introduction

Environmental concerns have been integrated into marketing practices for roughly 50 years, and green marketing has experienced an evolution from “ecological” green marketing, “environmental” green marketing, to “sustainable” green marketing (Peattie, 2001). Green consumption is of great significance to the sustainable development of humankind. A lot of countries have carried out active, practical exploration in the field of green consumption, such as legislation and environmental labeling certification (Prakash, 2002; Rahbar and Wahid, 2011; Sun et al., 2018). Many companies are also aware of the importance of green marketing and are working toward it. Nevertheless, consumers’ motivation to buy green products is not very high, due to insufficient information about the extent of the product’s greenness, lack of credibility regarding corporate claims, and the tendency to free-ride (Prakash, 2002). Therefore, how to promote consumers’ green consumption has become an important research agenda, and how to increase consumers’ willingness to buy green products has become a topic of discussion among many scholars.

Using anthropomorphism as a marketing tool can lead to many positive outcomes (Epley et al., 2007; Kim and McGill, 2011; Chen et al., 2016), including having a positive impact on the purchase intention of green products. Outlined in previous research, common anthropomorphic implementation strategies predominantly contain verbal communication (Ma et al., 2017), product design (Kim and McGill, 2011), and virtual spokesperson (Chen and Yang, 2017), and previous studies on the influence of anthropomorphic strategies on green behaviors mainly used verbal communication. For example, research has found that the anthropomorphism of nature leads people to feel more connected to nature, thus to show stronger use intention of green products (Tam et al., 2013). Recipients who have a strong need for effectance or social connection, anthropomorphic appeals motivate more conservation behavior and elicit more favorable message responses (Tam, 2015). As green consumption is a pro-environment and pro-social behavior, we argue that the use of anthropomorphic strategy will have a positive impact on the purchase intention of green products.

Eyes and expressions are often used to enhance the anthropomorphic effect. For example, the headlights and grilles of cars, the different modality of M & M bean villains, products or brands often use eyes or expressions to make their anthropomorphic image more vivid. Eye gaze has unique psychological properties; studies have shown that eye gaze increases pro-social motivation and induces more pro-social behaviors (Haley and Fessler, 2005; Bateson et al., 2006, 2013; Fathi et al., 2014). However, research has found that anthropomorphism can still be achieved in the absence of “eyes” (Xie and Wang, 2017). Therefore, it is unclear whether the anthropomorphic strategy with eyes has an impact on the purchase intention of green products or not. The main question examined in this study was whether the use of anthropomorphic strategies with or without images of eyes affects consumers’ willingness to buy green brands; if so, how.

In addition, anthropomorphic expressions have received a great deal of attention. The study found that participants were more likely to regard the car as a person and to rate it more positively when it presented a smiling face, and if the anthropomorphic facial expression of the product does not match the product positioning, the friendly expression will also lead to a conflict consumer perception (Aggarwal and McGill, 2007). Therefore, the effect of facial expressions of anthropomorphic products is uncertain. For example, for products with a higher luxury level, aggressive expressions are more favored by consumers than friendly ones. However, for products with lower luxury level, consumers would prefer products with friendly emoticons (Xie and Wang, 2017). There have been a large number of studies on the impact of facial expressions of anthropomorphic products on consumers. However, there are few studies that have specifically explored anthropomorphic effects by combining eyes and expressions, and the effect of the interaction between eyes and expressions on the purchase intention of green products in the anthropomorphic strategy is not clear.

To explore the question earlier, this research systematically examined the impact of anthropomorphic strategies with or without images of eyes, on consumers’ purchase intentions toward green brands. This research showed the positive effect of using anthropomorphic strategies with images of eyes compared with those without eyes on the purchase intention toward green products in the case of products that already contain anthropomorphic features through study 1. Study 2 shows that green trust mediates the relationship between anthropomorphic strategy and green brand purchase intention, with happy and sad expressions serving as boundaries for the anthropomorphic strategy. Finally, through study 3, we demonstrate the robustness of the experimental results and show that anthropomorphic strategies using images of eyes have a significant impact on green brand purchase intentions, whether through a virtual brand spokesperson or a product design.

## Literature Review and Research Hypotheses

### Anthropomorphism

In marketing, anthropomorphism refers to the tendency to attribute humanlike characteristics, intentions, and behaviors to non-human objects (Aggarwal and McGill, 2007; Epley et al., 2007). Using anthropomorphism as a marketing tool can lead to many positive outcomes, such as reducing consumers’ perceptions of risk (Kim and McGill, 2011), enhancing consumers’ perceptual fluency (Epley et al., 2007), and satisfying consumers’ need for social affiliation (Chen et al., 2016). As a result, more and more companies are starting to consciously anthropomorphize their products or brands. However, anthropomorphism does not always lead to positive effects, and many scholars have studied the negative effects of anthropomorphism and its mechanisms. For example, research has found that the presence of an anthropomorphized helper reduced game enjoyment during a computer game (Kim et al., 2016). Additionally, compared with non-anthropomorphic brands, anthropomorphic brands are more likely to elicit negative consumer attitudes toward the brand when it faces negative publicity caused by product wrongdoings (Puzakova et al., 2013). As a result, many scholars have explored which factors influence the way consumers used to respond to anthropomorphism from different perspectives. Among them, consumer characteristics and anthropomorphic implementation strategies are major concerns (Kim and Kramer, 2015; Chen et al., 2016).

As mentioned earlier, anthropomorphism has an impact on consumers’ attitudes and behaviors. How will anthropomorphism affect the purchase intention of green products? The study found that anthropomorphism can improve individuals’ pro-social behavior and promote one’s green purchase intention. Anthropomorphism enables individuals to associate products with their own similar characteristics (Kim and McGill, 2011), thus improves pro-social behavior toward others and doubles the donation amount (Burger et al., 2004). Similarly, research has found that the anthropomorphism of nature leads people to feel more connected to nature, which in turn promotes environmental conservation behavior (Tam et al., 2013). In addition, anthropomorphism makes individuals feel closer to the anthropomorphic object, which is more likely to stimulate green consumption behaviors (Nisbet et al., 2011).

### Impact of Eyes on a Customer’s Willingness to Buy Green Products

The eyes are the window to the mind and are an important representation for identifying the spiritual world. In facial recognition, the eyes tend to be the feature that people stare at the most (Bindemann et al., 2009). Both morphologically and functionally, eye gaze has unique psychological properties, and its psychological functions mainly include five aspects: providing information, maintaining interaction, expressing intimacy, social control, and service tasks (Lin, 2005). An extensive amount of empirical research suggests that eye gaze increases pro-social motivation (Fathi et al., 2014), inducing more pro-social behaviors (Bateson et al., 2013), such as reducing littering on campus and increasing the amount of charitable giving. Studies presented by Haley and Fessler (2005) and Bateson et al. (2006) have shown that both the presentation of eye images and eyelike images elicit pro-social behavior. It is worth mentioning that the eye effect on pro-social behavior has good validity, which has been confirmed in laboratory and field studies by many scholars (Haley and Fessler, 2005; Bateson et al., 2006, 2013; Oda et al., 2011).

In laboratory studies, Haley and Fessler (2005) found that in eyespots conditions, when two stylized eyelike shapes were displayed on a computer screen, participants who played the role of dictator distributed almost twice as much money to their partners as the those in the control group. Similarly, it was found that a picture of eyes displayed on participants’ computer screens acted as a cue for monitoring, thereby enhancing participants’ altruistic behavior (Mifune et al., 2010). The findings of Haley and Fessler (2005) are supported by Oda et al. (2011) study, which shows that eyes influence participants’ behavior by enhancing their expectations of rewards, in that participants expected their actions would enhance their reputation with third parties. In field experiments, Bateson et al. (2006) validated that images of eyes can elicit cooperative behavior in a real-world setting. They explored this by analyzing the number of customer payments that were made in an honesty box when different posters were hung in a café. They found that participants donated more money to boxes underneath a poster featuring a pair of eyes compared with the control image with pairs of flowers. In conclusion, people are more inclined to engage in altruistic and pro-social behavior when presented with images of eyes, which have a positive impact on the purchase intention of green products.

### Role of Eyes on Green Purchase Intention in Anthropomorphic Strategy

On the one hand, in the facial features of anthropomorphic products, eyes are one of the commonly used elements. For example, the decorative red strip of slot machines and the headlights of cars are manipulated as eyes (Kim and McGill, 2011; Xie and Wang, 2017) and anthropomorphized the computerized helper by adding humanlike facial features such as eyes (Kim et al., 2016). In addition, people will spontaneously personify objects with eyes (Haley and Fessler, 2005), and participants will feel being watched by presenting eyes images (Bateson et al., 2006). On the other hand, existing studies have found that when anthropomorphizing through facial features, subjects can still perceive different product appearance expressions in the absence of the “eye” element (Xie and Wang, 2017), i.e., the effect of anthropomorphizing without the “eye” can still be achieved. The product can be anthropomorphized by simply manipulating the arc geometry elements into the mouth (Xie and Wang, 2017). After reviewing the literature, what is not clear is whether eyes affect the effectiveness of product personification strategies. Specifically, this study focuses on whether eyes can improve the purchase intention of green products in the process of anthropomorphism influencing green purchase intention.

Based on the important role of eyes in facial recognition and the influence of eye effect on green purchase intention, this study suggests that in the process of anthropomorphic influence on green purchase intention, eyes can enhance the effect of anthropomorphic strategy on green product purchase intention. Both eyes and anthropomorphic strategies can promote pro-social behaviors, and the anthropomorphic strategies with eyes are more likely to enhance consumers’ motivation of pro-social behaviors, thus generating more positive purchase intention of green products. In addition, eyes, as an important facial feature, can enhance interaction and express intimacy (Lin, 2005). Therefore, this study suggests that eyes can enhance the effect of anthropomorphic strategy, although without eye element can make the interactive object perceive anthropomorphism (Xie and Wang, 2017). In conclusion, the researchers suggest that the anthropomorphic strategy with eyes can increase the willingness to buy green products. The following hypothesis is proposed:

H1: Anthropomorphic strategies featuring the use of images with eyes (vs. images without eyes) lead to more positive purchase intention of green products.

### Mediation Role of Green Trust

Green trust refers to “the willingness to rely on a product, service or brand based on the belief or expectation of its credibility, benevolence, and ability in terms of its environmental performance” (Chen, 2010), which emphasizes consumers’ trust in the environmental performance of products and trust willingness of companies’ green products or services, and believes that companies can fulfill their commitments (Chen and Chang, 2013). In green marketing, scholars have found that consumers can react badly to environmental marketing due to false, unsubstantiated, or exaggerated claims (Carlson et al., 1993; Sun et al., 2020). The credibility of the information presented to consumers is a key factor influencing whether green marketing is effective (Prakash, 2002) because consumers’ skepticism toward green claims will lead to their negative attitude toward green products (Chang, 2011). Similarly, consumers’ trust in eco-labels and eco-brands positively affects their purchase intentions and actual purchase behavior (Nik Abdul Rashid, 2009; Rahbar and Wahid, 2011). Empirical studies from different countries also show that there is a significant positive relationship between consumers’ trust in green brands and their willingness to use green products (Alshura and Zabadi, 2016). In short, when companies carry out green marketing activities, green trust will influence consumers’ green purchase intention (Sheng and Lin, 2018).

The presentation of eyes will increase the perception of trust; for example, adding a virtual agent with head and eyes will increase user’s perception of trustworthiness and friendliness toward the virtual communication object (Donath, 2007). In addition, using a set of three dots resembling the watching eyes weak social cues in investment gaming improves interpersonal trust and trust-based decision-making, demonstrating the important role of eye cues in enhancing interpersonal trust (Xin et al., 2016). In the marketing process of green products, anthropomorphic strategies will urge consumers to judge products in a humanlike way (Aggarwal and McGill, 2007; Xie and Wang, 2017). Therefore, presenting eyes can provide interpersonal information (Lin, 2005) and convey trustworthy social clues, thus improving consumers’ trust in green attributes. Therefore, this study argues that anthropomorphic strategies with eyes increase consumers’ green trust in products and thus positively influence green purchase intentions. From this, the following hypothesis is proposed:

H2: An anthropomorphic strategy that uses eyes enhances consumers’ green trust in green products, thus increasing consumers’ willingness to buy.

### Moderating Effect of Facial Expressions

Facial expressions play a very important role in emotional communication and interpersonal interactions. Although emotion theorists and behavioral ecologists have different views on facial expressions, studies have shown that facial expressions can convey both emotional states and feelings, intentions, and wishes (Horstmann, 2003). Knutson (1996) validated that expressions of emotion (e.g., anger, disgust, fear, happiness, and sadness) can convey interpersonal messages, and Aggarwal and McGill (2007) found that expressions found on products influence consumer attitudes. Further, some scholars have confirmed that the “expressions” of products, like those in interpersonal interactions, affect the judgment of the interacting subjects (Xie and Wang, 2017).

Ekman (1993) identified anger, disgust, fear, happiness, and sadness as the basic emotional facial expressions, whereas Marin et al. (2006) classified the virtual character’s emotions like joy, distress, pity, boredom, and fear. We selected happy facial expression and sad facial expression, the two most common expressions, as study variables. On the one hand, happy facial expression increases ratings of familiarity (Baudouin et al., 2000). Also, Aggarwal and McGill (2007) argue that a smile is more congruent with the general human schema than a frown. On the other hand, scholars have found that sad facial expression can awaken empathy, enhance individuals’ willingness to give charitably (Fisher et al., 2008), and increase people’s spending while shopping (Lerner et al., 2004). When people perceive other’s sadness, their behavior and judgments will change dramatically (Marsh et al., 2003). Although there have been several previous studies exploring facial expressions found on products, according to our research, it is still inconclusive whether happy or sad facial expressions found on anthropomorphic products have a more significant effect on consumers’ willingness to buy. Additionally, there is no specific study combining facial expressions with eyes in anthropomorphism strategies.

Facial expression processing is based on communication signals, such as eye gaze (Adams and Kleck, 2003). Therefore, the presence of eyes can affect the processing of a facial expression. However, experiments by Boucher and Ekman (1975) demonstrate that no one region of the face best reveals emotions and that different facial regions disclose different emotions. For example, the eyes are most important for expressing sadness, whereas the mouth is most important for expressing happiness. Calvo et al. (2014) study also show that happy expressions are mainly identified through the mouth. Therefore, in the happy facial expression of anthropomorphic products, the effect of eyes on the processing of interpersonal information of product expression is weak. However, the presentation of eyes is very important for consumers to process the interpersonal information of sad facial expressions of products. This study argues that the presence or absence of eyes interacts with facial expressions and influences consumers’ purchase intentions. Specifically, the following hypotheses are proposed:

H3a: In the product anthropomorphism feature, when sad facial expressions are present, anthropomorphic strategies with images of eyes (vs. without eyes) promote higher purchase intention of green products.
H3b: In the product anthropomorphism feature, when happy facial expressions are present, anthropomorphic strategies with images of eyes (vs. without eyes) do not significantly affect the purchase intentions of green products.

**FIGURE 1:**
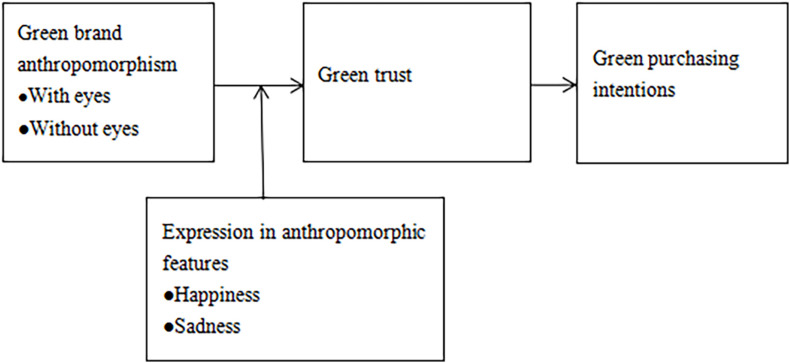
Research framework.

The theoretical framework of this study is shown in Figure 1.

## Materials and Methods

In this study, three studies were conducted to test the earlier hypotheses. Study 1 aims to explore whether the presence of eyes can improve the effect of anthropomorphic strategy on the purchase intention of green products and the mediation role of green trust. Study 2 aims to examine further the moderation role of expression in anthropomorphic facial features. Finally, study 3 replicated the findings in study 1 and study 2 using different anthropomorphic experimental materials, which verified the robustness of the experimental results.

### Study 1

In study 1, researchers presented anthropomorphic green products by advertising slogans and image stimuli to examine the different effects of anthropomorphic strategies with and without eyes on consumers’ green purchase intentions and their mechanisms of action. This study predicted that the use of anthropomorphic strategies containing images with eyes in brands’ anthropomorphic features would lead to more positive green brand purchase intentions.

#### Design, Stimuli, and Procedure

In study 1, sample collection was conducted through an online platform, and a total of 216 subjects were chosen to participate in the study. Excluding the five samples that were disrupted by environmental interference, the total valid samples obtained were 211 (95 females; *M*_age_ = 27.31, SD_age_ = 9.30).

**FIGURE 2:**
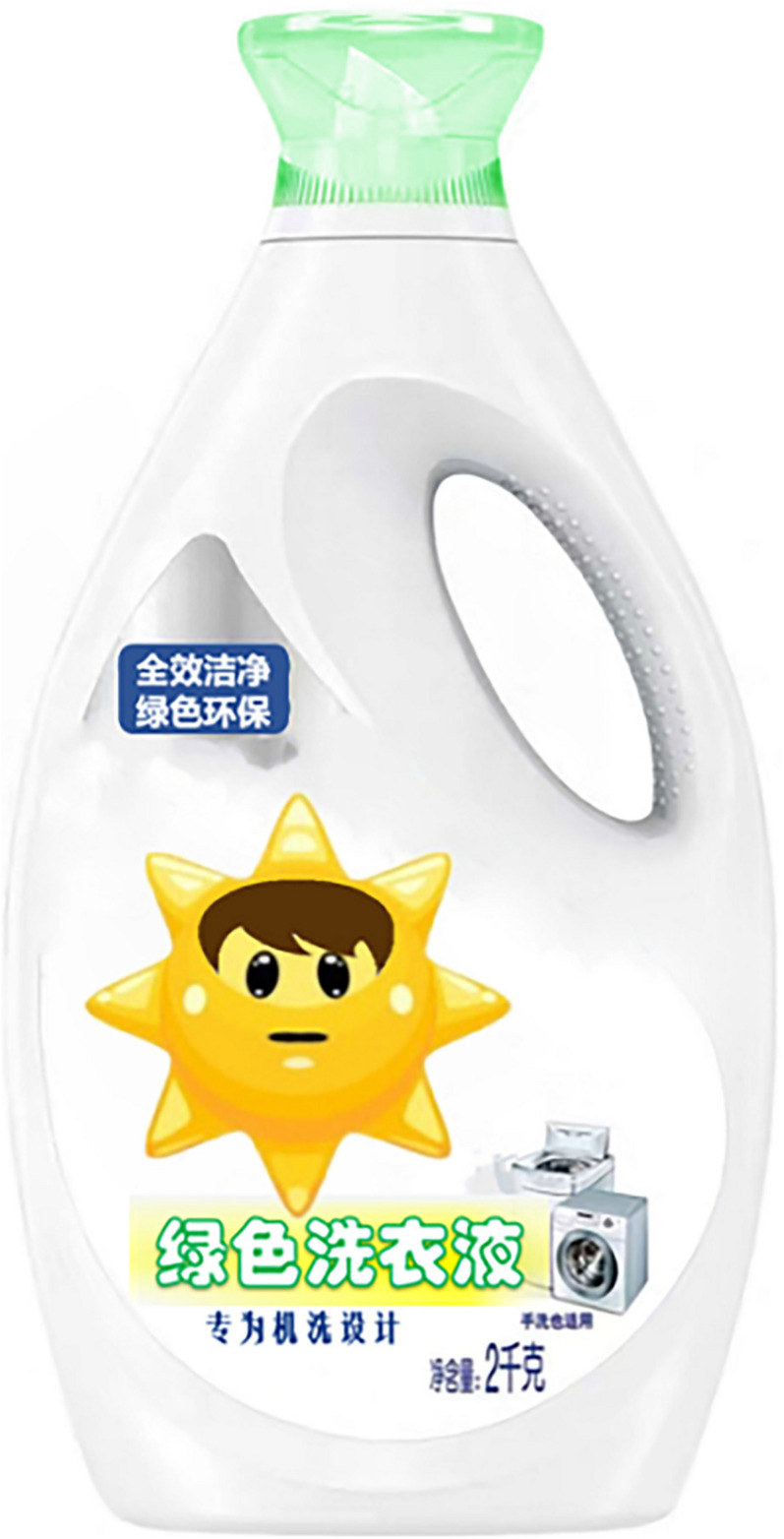
Anthropomorphic strategy with eyes.

**FIGURE 3:**
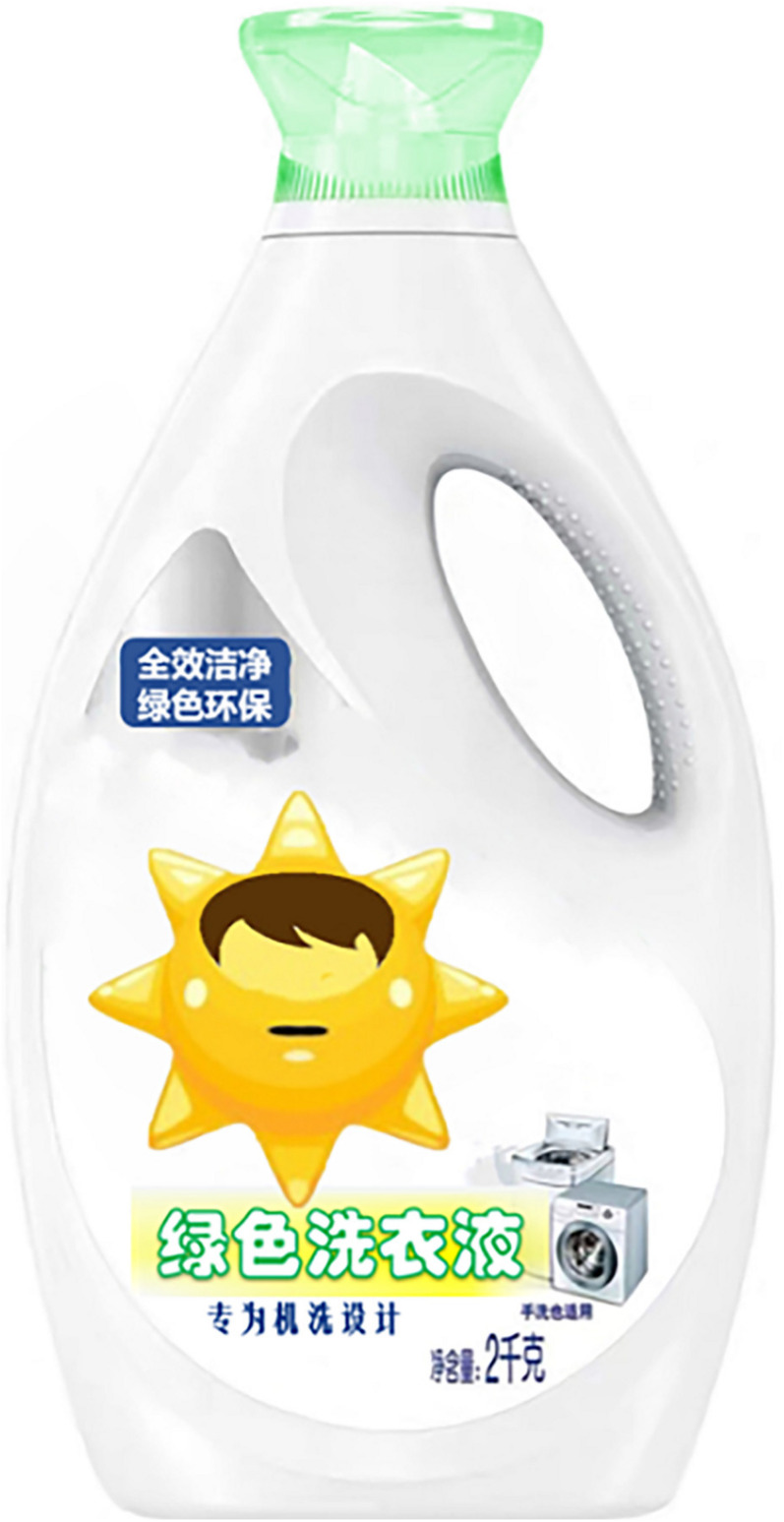
Anthropomorphic strategy without eyes.

The study used a one-factor (an anthropomorphic strategy with eyes vs. without eyes) between-subjects design, and participants were randomly assigned to either the group for the anthropomorphic strategy with eyes or to the group for the anthropomorphic strategy without eyes. To avoid consumers being directly influenced by familiar brands, the researchers used virtual brands and designed advertising slogans and product images. First, both groups of participants read the same green laundry detergent ad (see Appendix A). Participants were then shown pictures of the corresponding green laundry detergent products (as shown in Figures 2,3). Next, participants were asked to describe their willingness to buy the product as well as feelings of green trust and did the anthropomorphic manipulation check. These were all done via a seven-point Likert scale. Finally, subjects filled out demographic information.

#### Measures

First, this study referred to the scale developed by Lao (2013) (α = 0.785) to measure green purchasing intentions. Participants were asked how strongly they agree or disagree with the following statements: (1) I would be willing to collect and learn more about green laundry detergent; (2) I would recommend that my relatives and friends purchase green laundry detergent; (3) I would show and recommend green laundry detergent to my family; and (4) I would purchase green laundry detergent if I needed to (1 = strongly disagree; 7 = strongly agree). Next, participants’ measure of green trust was assessed (Chen, 2010) (α = 0.797). They were asked how strongly they agree or disagree with the following statements: (1) You feel that this brand’s environmental commitments are generally reliable; (2) You feel that this brand’s environmental performance is generally dependable; (3) You feel that this brand’s environmental argument is generally trustworthy; (4) This brand’s environmental concern meets your expectations; and (5) This brand keeps its promises and commitments regarding environmental protection (1 = strongly disagree; 7 = strongly agree). For the anthropomorphic manipulation check, the subjects were asked to answer on a seven-point Likert scale (1 = not very much; 7 = very much) how much they associated the product they had just seen with a person (Wang et al., 2014).

#### Results and Discussion

##### Manipulation check

The results of the data showed that the anthropomorphic manipulation was successful (*M*_with eyes_ = 5.60, SD = 1.10 vs. *M*_without eyes_ = 5.49, SD = 1.11, *t*(209) = 0.760, *p* = 0.448) and there was no significant difference between the two in terms of anthropomorphic degree.

##### Willingness to buy green products

**FIGURE 4:**
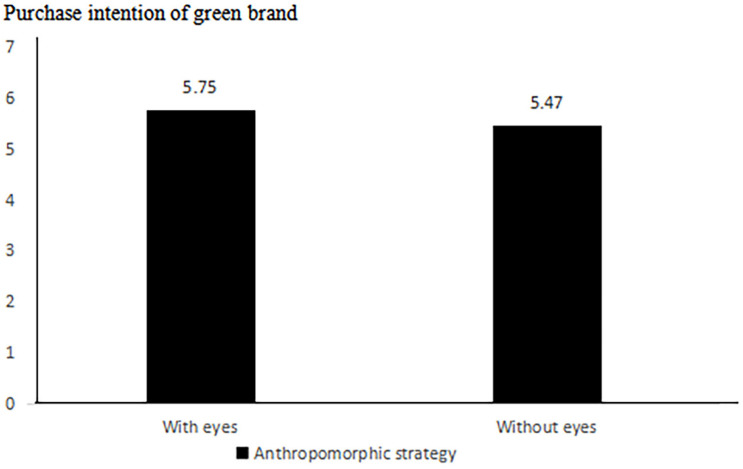
Impact of anthropomorphic strategies on green brand purchase intention.

The researchers found (as shown in Figure 4) that the presence of eyes (the anthropomorphic strategy) has a significant effect on purchase intentions for green brands using an independent sample *t*-test. The purchase intentions for green products in the group of anthropomorphic strategies with eyes were significantly higher than anthropomorphic strategies without eyes (*M*_with eyes_ = 5.75, SD = 0.81 vs. *M*_without eyes_ = 5.47, SD = 0.90, *t*(209) = 1.981, *p* < 0.05), thus supporting Hypothesis 1.

##### Mediation analysis

**FIGURE 5:**
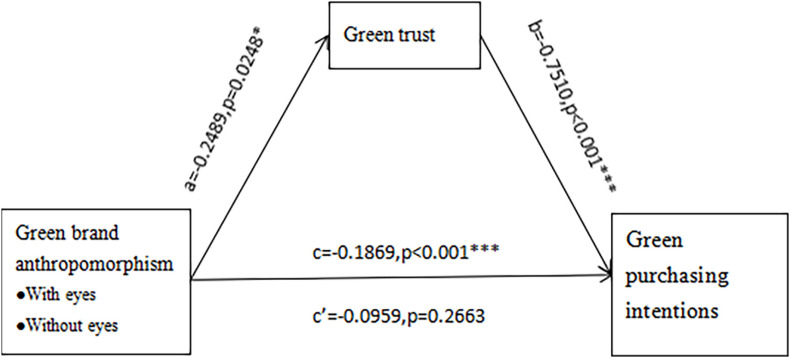
Mediation effect of green trust. *p < 0.05, ***p < 0.001.

To further analyze the potential mechanisms through which anthropomorphic strategies affect consumers’ green product purchase intentions, we conducted a mediation analysis (Model 4; bias-corrected bootstraps = 5,000; Hayes, 2013) using PROCESS. The results showed that the direct effect of anthropomorphic strategies with and without eyes on consumers’ willingness to buy green products was not significant (95% confidence interval [CI] [−0.2656, 0.0737]), and the indirect effect was significant (95% CI [−0.3731, −0.0332]). Consistent with Hypothesis 2, green product trust fully mediated the effect of anthropomorphic strategies with or without eyes on consumers’ green product purchase intentions. Figure 5 shows the mediating effect path between green brand anthropomorphism with or without eyes and green brand purchase intention.

Study 1 found that among product anthropomorphic features, compared with anthropomorphic strategies without eyes, anthropomorphic strategies with eyes led to more positive green purchase intentions, which are mediated by green trust.

In the next study, this paper continues to explore the boundary conditions for the discussed effects. We will check the interaction effect of eyes and emotional facial expression on green purchase intentions. We hypothesize that in the product anthropomorphism feature, when sad facial expressions are present, anthropomorphic strategies with images of eyes (vs. without eyes) promote higher purchase intention of green products; however, the effect is not significant in the condition of happy facial expressions.

### Study 2

#### Design, Stimuli, and Procedure

**FIGURE 6:**
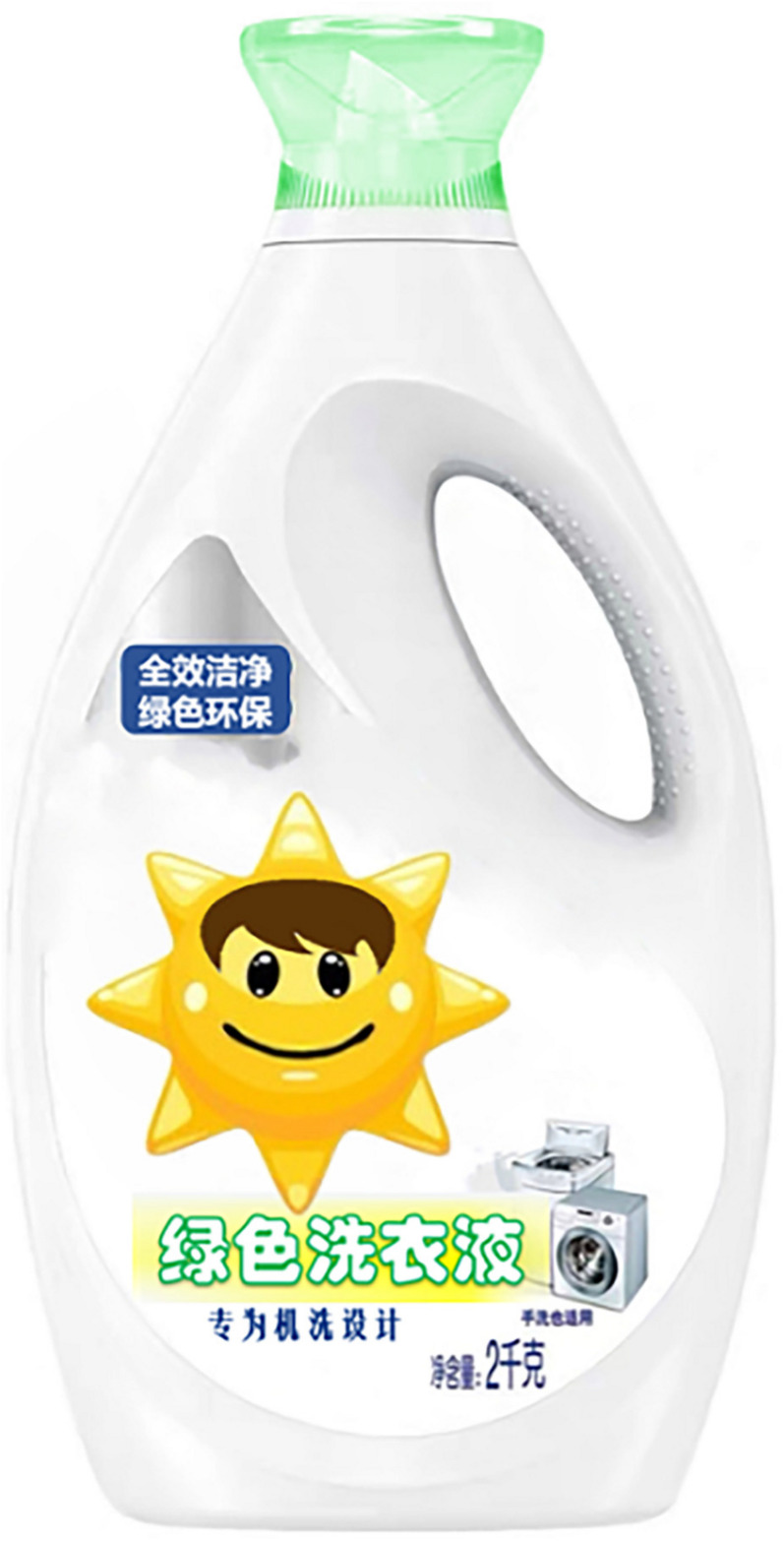
Anthropomorphic strategy of happy expressions with eyes.

**FIGURE 7:**
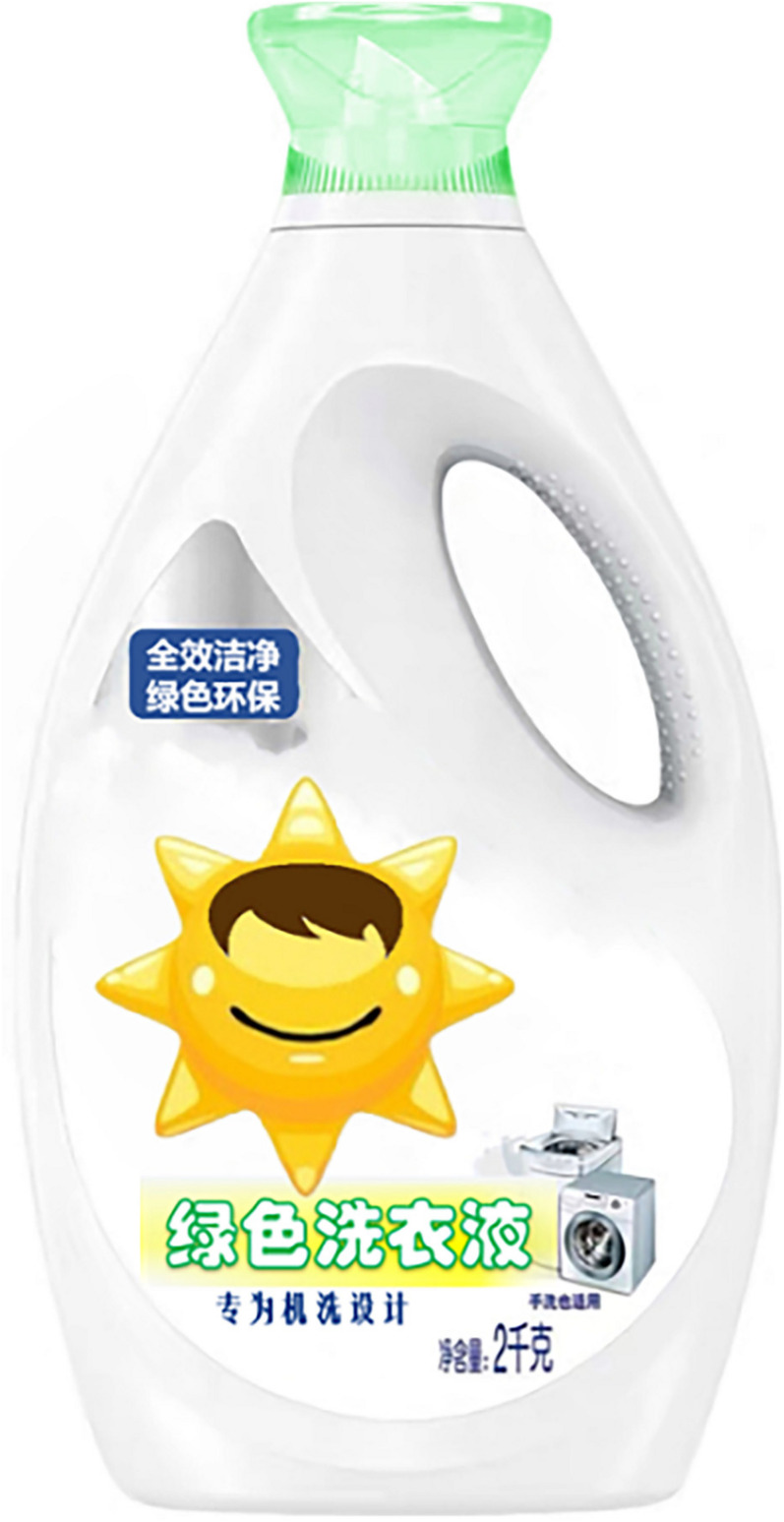
Anthropomorphic strategy of happy expressions without eyes.

**FIGURE 8:**
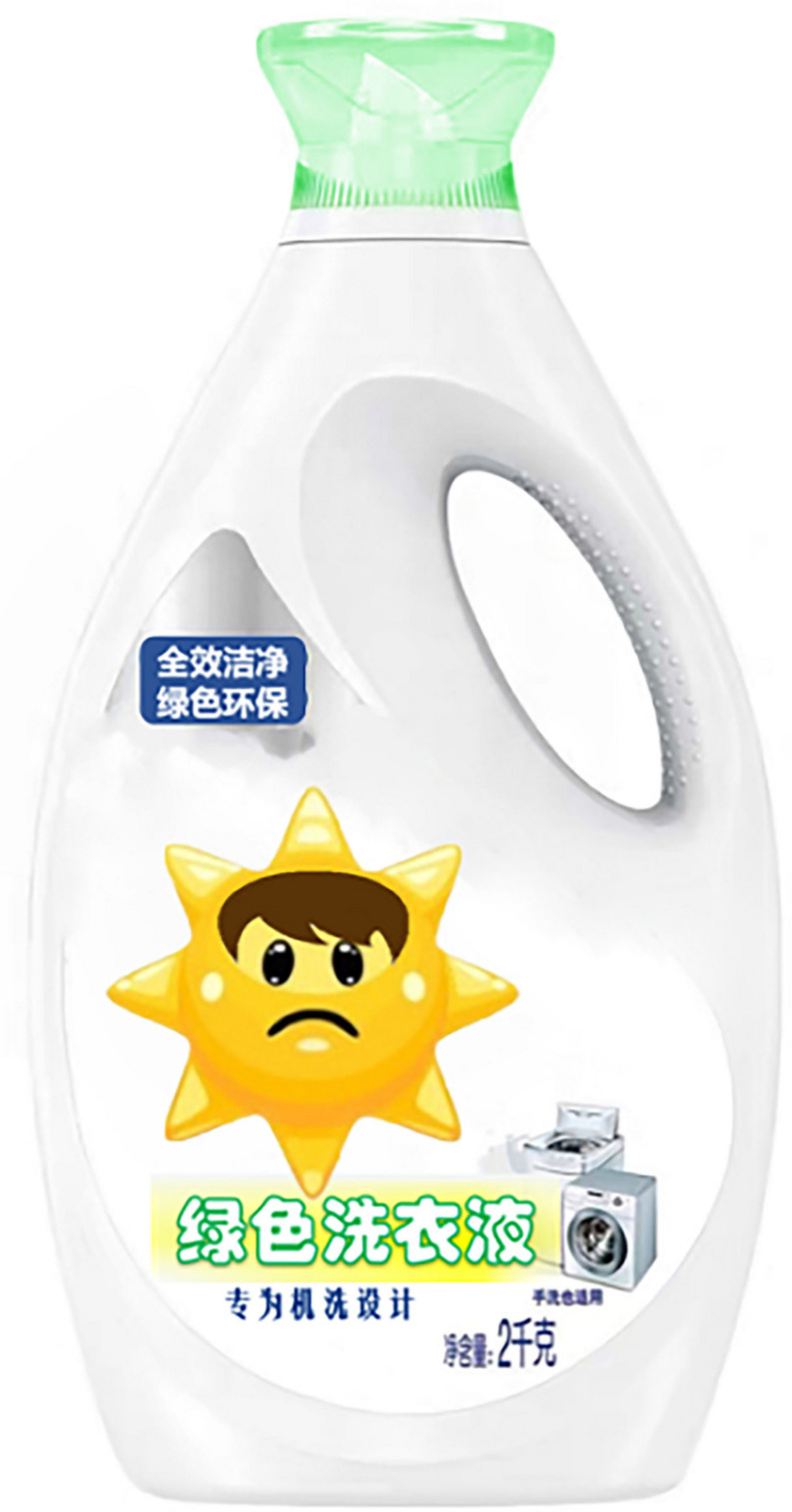
Anthropomorphic strategy of sad expressions with eyes.

**FIGURE 9:**
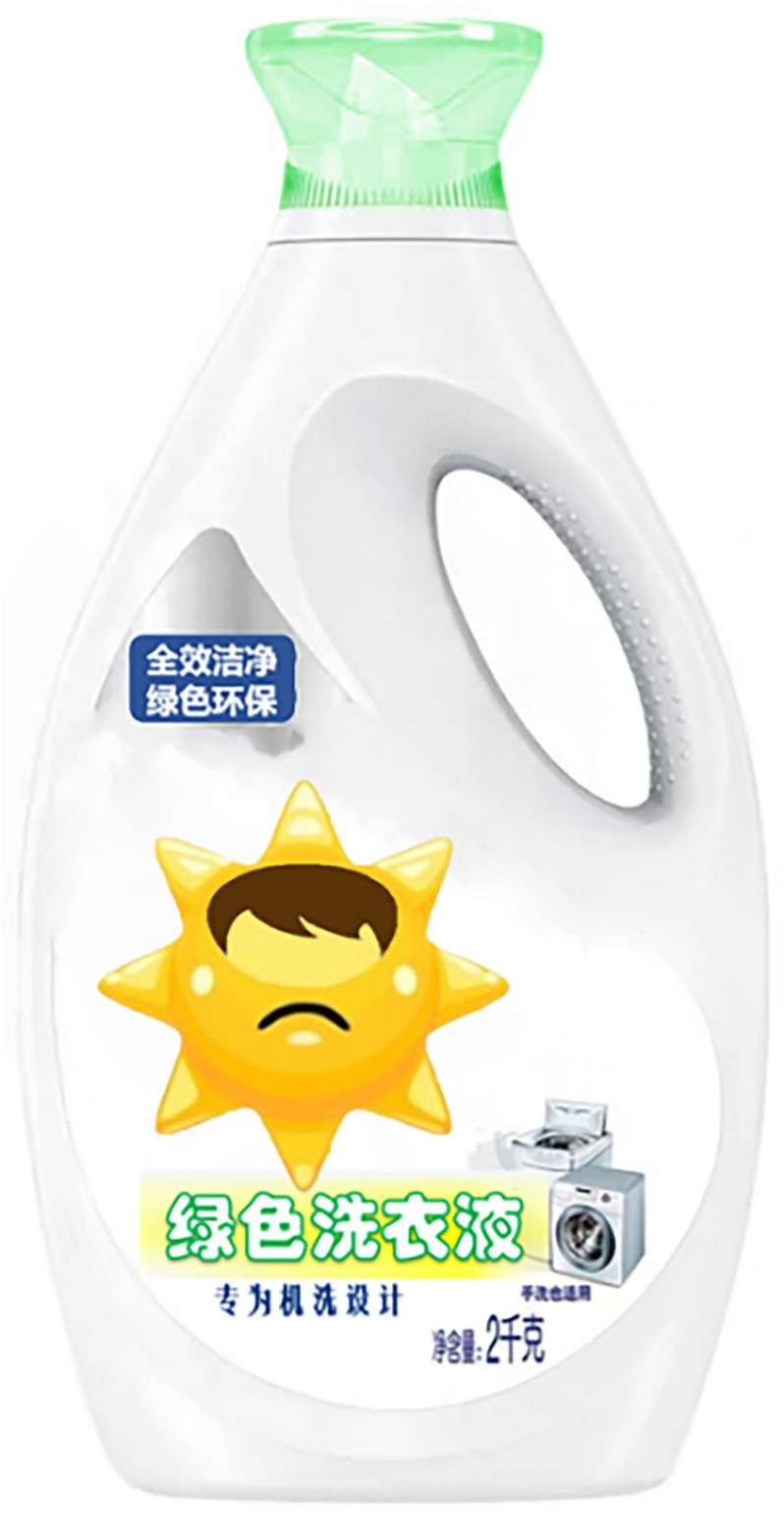
Anthropomorphic strategy of sad expressions without eyes.

In study 2, a total of 400 subjects participated in the study. Excluding the 10 samples that were disrupted due to environmental interference, the final valid sample obtained was a total of 390 (198 females; *M*_age_ = 28.32, SD_age_ = 8.16). Study 2 used a 2 (anthropomorphic strategy: with eyes vs. without eyes) × 2 (facial expression: sadness vs. happiness) between-subject design in which participants were randomly assigned to any one of four experimental groups. To avoid consumers being directly influenced by familiar brands, the researchers used virtual brands and designed advertising slogans and product images. Participants read the same green laundry detergent ad as in study 1 (see Appendix A) and were then shown pictures of the corresponding green laundry detergent products (as shown in Figures 6–9). Further, to ensure that differences in emotions did not drive participants’ evaluations due to the different expressions, the possible confounding effects of emotions were examined (α = 0.821) (Hagtvedt, 2011). Further, participants were asked to describe their willingness to buy green products, their feelings about green trust, and their anthropomorphic manipulation was checked. All of these data were captured via a seven-point Likert scale. Finally, subjects performed an expression recognition check and filled in demographic information.

#### Measures

As in study 1, participant’s willingness to buy green was measured on a scale developed by Lao (2013) (α = 0.814), and their green trust was measured on a scale developed by Chen (2010) (α = 0.775). For the anthropomorphic manipulation check, the subject was asked to answer on a seven-point Likert scale (1 = not very much; 7 = very much) how much they associated the product just seen with a person. To test the manipulation of expressions, subjects were asked to indicate on a scale whether they perceived the stimulus as happy or sad (Landwehr et al., 2011).

#### Results and Discussion

##### Manipulation check

Results showed that anthropomorphic manipulation was successful (*M*_with eyes_ = 5.35, SD = 1.02 vs. *M*_without eyes_ = 5.29, SD = 1.09, *t*(388) = 0.550, *p* = 0.583) and there was no significant difference between the two in terms of anthropomorphic degree. As expected, subjects assigned to different anthropomorphic strategic expressions did not differ significantly on the mood check items (*M*_happy_ = 5.10, SD = 1.03 vs. *M*_sad_ = 5.03, SD = 1.09, *t*(388) = 0.613, *p* = 0.540), ruling out the idea that different moods add to the manipulation of the consumer. In addition, participants in the happy facial expression group and those in the sad facial expression group identified each expression correctly.

##### Willingness to buy green products

**FIGURE 10:**
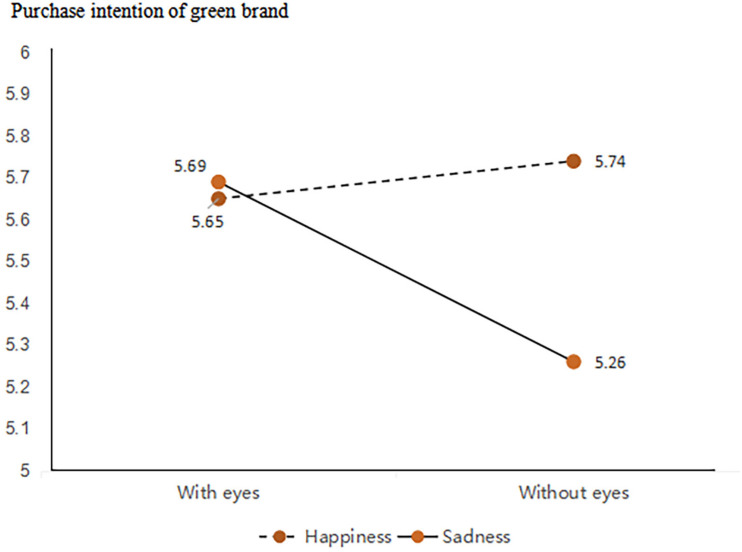
Moderating role of expression.

We conducted a two-way ANOVA and simple effect analysis. The results of the two-way ANOVA showed an interacting effect between anthropomorphic strategies with and without eyes and the expressions of anthropomorphic features (*F*(1,386) = 7.16, *p* < 0.05). Simple effect revealed that anthropomorphic strategy with eyes would lead to higher purchase intentions of green products when the expression is sad (*M*_with eyes_ = 5.69, SD = 0.84 vs. *M*_without eyes_ = 5.26, SD = 1.07, *F*(1,386) = 9.03, *p* < 0.05). In contrast, participants’ willingness to buy green products did not differ between anthropomorphic strategy with eyes vs. without eyes group when the expression is happy (*M*_with eyes_ = 5.65, SD = 0.94 vs. *M*_without eyes_ = 5.74, SD = 0.94, *F*(1,386) = −0.45, *p* = 0.503). Results support Hypothesis 3 (as shown in Figure 10).

##### Moderated mediation analysis

We conducted a moderated mediation analysis (Model 8; bias-corrected bootstraps = 5,000; Hayes, 2013) using PROCESS. The anthropomorphic strategy is used as an independent variable and the consumer’s willingness to buy green products as a dependent variable. Green trust is used as a mediating variable and the anthropomorphic strategy’s expression as a moderating variable. The results reveal that the moderated mediation effect was significant (95% CI [−0.5756, −0.0726]). Specifically, the mediating effect of green trust was not significant for happy facial expressions (95% CI [−0.1244, 0.2455]) and was significant for sad facial expressions (95% CI [−0.4323, −0.0908]) among the product anthropomorphic features.

To verify the robustness of the results and to broaden the range of product anthropomorphic features (from product pattern anthropomorphism to product design anthropomorphism), a more representative green air conditioner (Lao, 2013) was selected as a stimulus for study 3.

### Study 3

#### Design, Stimuli, and Procedure

**FIGURE 11:**
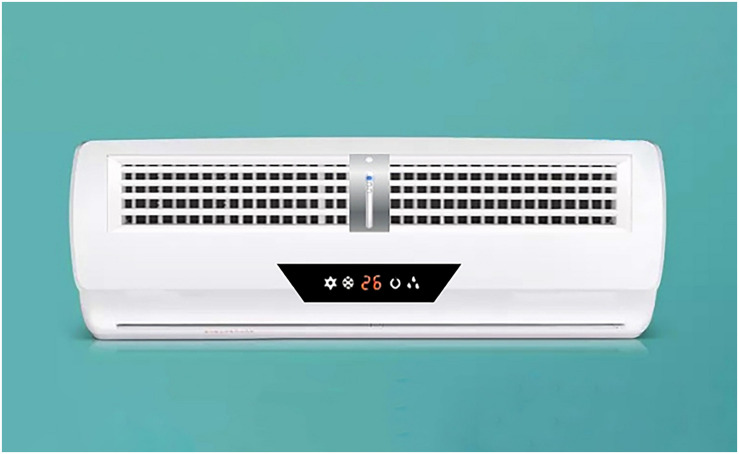
Anthropomorphic strategy of happy expressions with eyes.

**FIGURE 12:**
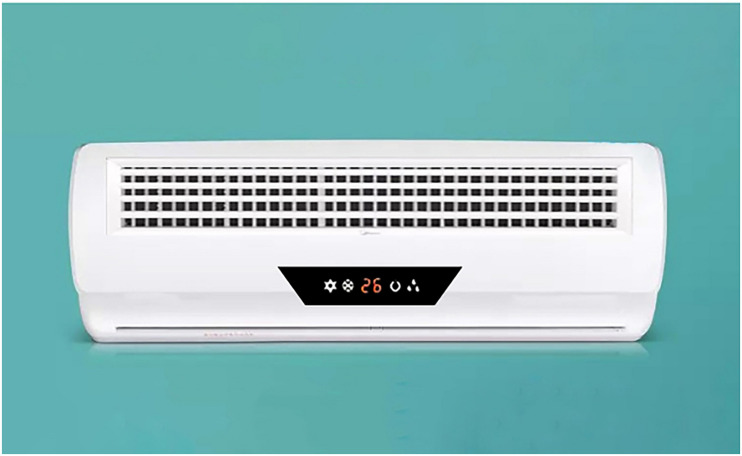
Anthropomorphic strategy of happy expressions without eyes.

**FIGURE 13:**
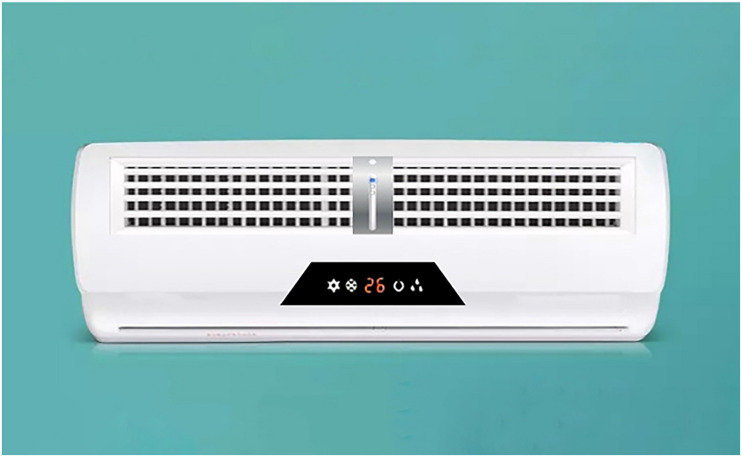
Anthropomorphic strategy of sad expressions with eyes.

**FIGURE 14:**
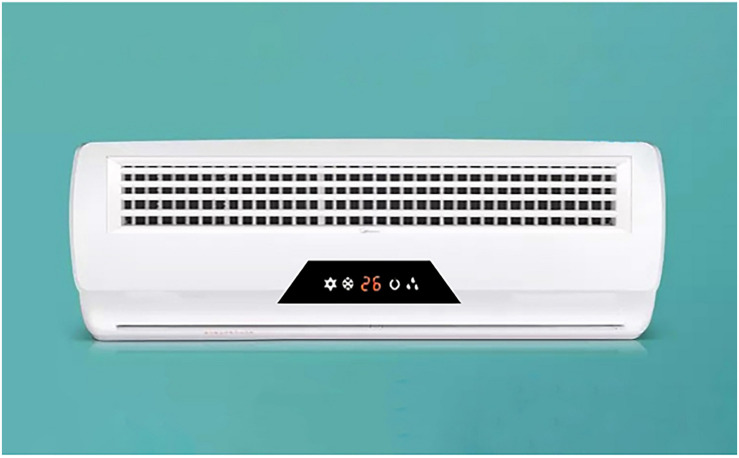
Anthropomorphic strategy of sad expressions without eyes.

In study 3, a total of 450 subjects participated in the study. Excluding the 14 samples that were disrupted due to environmental interference, a total of 436 valid samples were obtained (196 females; *M*_age_ = 29.35, SD_age_ = 9.47). This study used a 2 (anthropomorphic strategy: with eyes vs. without eyes) × 2 (expression: sadness vs. happiness) between-subject design in which participants were randomly assigned to any one of four experimental groups. To avoid consumers being directly influenced by familiar brands, the researchers used virtual brands and designed advertising slogans and product images. First, participants read the green air conditioner ad (see Appendix B). The participants were then shown pictures of the corresponding green air conditioning products (as shown in Figures 11–14). The presence or absence of eyes, as well as the happy or sad expressions, was manipulated using the outlet and rectangular bars, respectively. Next, to ensure that differences in emotions did not drive participants’ evaluations due to the expressions they saw, the possible confounding effects of emotions were examined (α = 0.724) (Hagtvedt, 2011). Additionally, participants were asked to describe their willingness to buy green products and feelings about green trust. Anthropomorphic manipulation checks were also performed. This was all done via a seven-point Likert scale. Finally, subjects performed an expression recognition check and filled in demographic information.

#### Measures

As in studies 1 and 2, the green purchase intention scale (α = 0.802) developed by Lao (2013) was drawn on as well as and the green trust scale (α = 0.815) developed by Chen (2010). For the anthropomorphic manipulation check, the subjects were asked to answer on a seven-point Likert scale (1 = not very much; 7 = very much) how much they associated the product just seen with a person. To test the manipulation of expressions, subjects were asked to indicate on a scale whether they perceived the stimulus as sad or happy (Landwehr et al., 2011).

#### Results and Discussion

##### Manipulation check

The data results showed that anthropomorphic manipulation was positive (*M*_with eyes_ = 5.41, SD = 1.00 vs. *M*_without eyes_ = 5.38, SD = 1.10, *t*(434) = 0.310, *p* = 0.757) and there was no significant difference between the two in terms of anthropomorphic degree. As expected, subjects assigned to different anthropomorphic strategic expressions did not differ significantly on the mood check items (*M*_happy_ = 5.27, SD = 0.81 vs. *M*_sad_ = 5.14, SD = 0.89, *t*(434) = 1.477, *p* = 0.140), ruling out the idea that different moods add to the manipulation of the consumer. In addition, participants in the happy facial expression group and those in the sad facial expression group identified each expression correctly.

##### Willingness to buy green product

**FIGURE 15:**
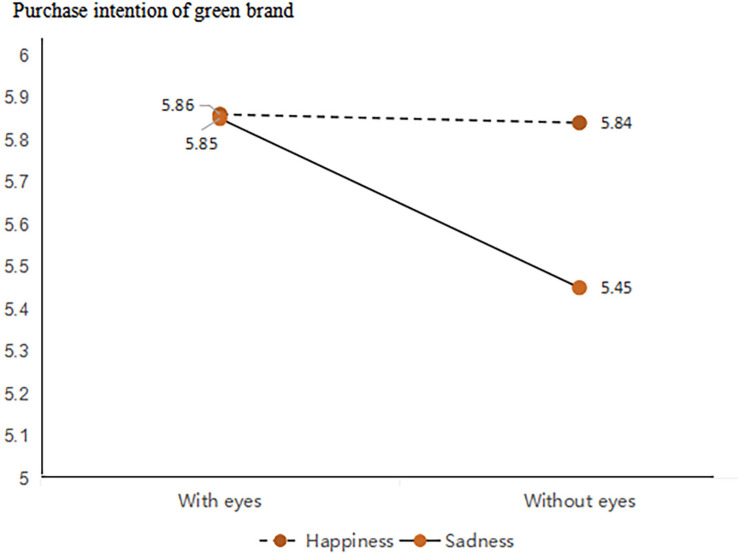
Moderating role of expression.

We conducted a two-way ANOVA and simple effect analysis. The results of the two-way ANOVA showed an interaction between anthropomorphic strategies with and without eyes and the expressions of anthropomorphic features (*F*(1,432) = 4.61, *p* < 0.05). Simple effect revealed that in the happy facial expression condition, the effect of the product featuring eyes on green purchase intentions was not significant (*M*_with eyes_ = 5.86, SD = 0.09 vs. *M*_without eyes_ = 5.84, SD = 0.09, *F*(1,432) = 0.04, *p* = 0.85); however, in the sad facial expression condition, the effect of the presence of eyes on green purchase intentions was significant (*M*_with eyes_ = 5.85, SD = 0.83 vs. *M*_without eyes_ = 5.45, SD = 1.18 *F*(1,432) = 10.52, *p* < 0.05). Results support Hypothesis 3 (as shown in Figure 15).

##### Moderated mediation analysis

We conducted a moderated mediation analysis (Model 8; bias-corrected bootstraps = 5,000; Hayes, 2013) using PROCESS. The anthropomorphic strategy is used as an independent variable and the consumer’s willingness to buy green products as a dependent variable. Green trust is used as a mediating variable and the anthropomorphic strategy’s expression as a moderating variable. The results reveal that the moderated mediation effect was significant (95% CI [−0.4904, −0.0065]). Specifically, the mediating effect of green trust was not significant for happy facial expressions (95% CI [−0.1385, 0.1557]) and was significant for sad facial expressions (95% CI [−0.4273, −0.0585]) among the product anthropomorphic features.

Study 3 replicated the findings in study 1 and study 2 using different anthropomorphic experimental materials, which enhanced the robustness of the experimental results.

## General Discussion

In green marketing, how to enhance consumers’ willingness to buy green brands is a common concern of scholars as well as a problem in green marketing practices. Previous studies have focused on the demographic characteristics, psychological factors, and psychological mechanisms of green consumption behavior. However, according to the current research, no in-depth studies have been conducted from an anthropomorphic perspective. Using anthropomorphism as a marketing tool has many positive effects for businesses, brands, or products (Epley et al., 2007; Kim and McGill, 2011; Chen et al., 2016). Therefore, this study combines anthropomorphic strategies with green marketing to explore the impact of anthropomorphic strategies with or without images of eyes on green purchase intentions. This study expands the perspective of green marketing and provides new ideas for increasing consumers’ willingness to buy green brands.

Study 1 found that among product anthropomorphic features, anthropomorphic strategies with images of eyes (vs. images without eyes) led to more positive green product purchase intentions, as anthropomorphic strategies with images of eyes increased consumers’ green trust and thus green brand purchase intentions. Many literature have focused on the effects of anthropomorphism on consumers (Epley et al., 2007; Kim and McGill, 2011; Chen et al., 2016); however, little researches have been done to combine eyes and facial features with anthropomorphism. Our study further enriches and deepens the field of anthropomorphism. Further, study 2 targeted the moderating factors that influence consumers’ willingness to buy green brands, further revealing the boundary conditions that underlie the earlier discussed findings. Among the product anthropomorphic features, anthropomorphic strategies with or without images of eyes were not significant for green brands’ purchase intentions when happy expressions were presented, whereas anthropomorphic strategies with images of eyes (vs. images without eyes) were more positive for green brands’ purchase intentions when sad expressions were presented. Although there have been a number of previous studies exploring anthropomorphic product expressions (Aggarwal and McGill, 2007; Xie and Wang, 2017), there have been few studies that explore the effect of combining images of eyes and facial expressions on consumers’ purchase intentions, and our study explores this gap. Finally, study 3 extended the brand anthropomorphism feature from product packaging anthropomorphism to broader product design anthropomorphism, verifying the robustness of the experimental results. Therefore, this study provides a reference for future research on green brand anthropomorphism.

This study examines the positive impact of anthropomorphic implementation strategies on green brand purchase intentions from the perspective of anthropomorphic facial features and also makes recommendations for firms’ marketing campaigns. Firstly, the researcher reminds companies that they can use anthropomorphic strategies for green marketing campaigns. When conducting product design, companies can manipulate the perceived facial areas and facial expressions of consumers through product appearance or product packaging, and so on. The use of anthropomorphic strategies with images of eyes can reduce consumers’ guardedness and suspicion, thereby increase their willingness to buy green products. Secondly, this study found that the interaction effect of facial expressions in anthropomorphic features and anthropomorphic strategies with or without images of eyes affected consumers’ willingness to buy green. Companies can determine the product’s expression based on the actual needs of the product or brand and then design anthropomorphic facial features.

This study explores the impact of different anthropomorphic strategies on consumers’ green brand purchase intentions, enriching and deepening the research in the field of green marketing. However, there are also limitations to this study. Also, in the future, the researcher hopes to conduct a more in-depth study in the following areas. First, product facial expressions do not only include happy and sad (Marin et al., 2006); this study explores only two more typical facial expressions, and other facial expressions in green product anthropomorphism can be explored in the future. Secondly, whether product anthropomorphism of other facial areas or limbs other than the eyes affects green product purchase intentions is also a research question worth exploring. In addition, due to limited resources, the sample for this study was selected from China. Ekman et al. (1987) noted that people interpret facial expressions differently in different cultural contexts. Future research could consider starting with the dimension of cultural variables to check whether facial expressions in brand anthropomorphism have the same effect on consumers in different cultures.

## Data Availability Statement

The raw data supporting the conclusions of this article will be made available by the authors, without undue reservation.

## Ethics Statement

Ethical review and approval was not required for the study on human participants in accordance with the local legislation and institutional requirements. Written informed consent from the participants was not required to participate in this study in accordance with the national legislation and the institutional requirements.

## Author Contributions

ZT and QZ contributed to the conception and design of the study. ZT organized the database. TL performed the statistical analysis. TL and JF wrote the first draft of the manuscript. QZ wrote sections of the manuscript. All authors contributed to manuscript revision, read and approved the submitted version.

## Conflict of Interest

The authors declare that the research was conducted in the absence of any commercial or financial relationships that could be construed as a potential conflict of interest.

## Appendix

### A. Study 1 and Study 2: Green Laundry Detergent Ad

The following product is a non-toxic, healthy, and skin-friendly green laundry detergent. The product has strong stain removal qualities and can effectively remove stubborn stains such as oil stains, sweat stains, juice residues, and others. Compared with similar products, this laundry detergent is water-saving and electricity-saving and consumes only 50% of the total energy of ordinary laundry detergent, making it a low-carbon, environmentally friendly green product. However, this green laundry detergent is 20% more expensive than regular laundry detergent.

### B. Study 3: Green Air Conditioning Ads

The following product is an air conditioner made from recycled materials. It is sustainably produced and creates no pollution in the manufacturing process. The air conditioner uses energy-saving technology and consumes 50% of the energy of conventional air conditioners on the market. The air conditioner has a good combination of performance and longevity in accordance with national standards. Moreover, the air conditioner can be recycled, making it a low-carbon, environmentally friendly green product. However, this green air conditioner is 20% more expensive than a regular air conditioner.
